# Early and accurate detection and diagnosis of heart disease using intelligent computational model

**DOI:** 10.1038/s41598-020-76635-9

**Published:** 2020-11-12

**Authors:** Yar Muhammad, Muhammad Tahir, Maqsood Hayat, Kil To Chong

**Affiliations:** 1 Department of Computer Science, Abdul Wali Khan University Mardan, Mardan, 23200 KP Pakistan; 2 Department of Electronic and Information Engineering, Jeonbuk National University, Jeonju, 54896 South Korea

**Keywords:** Cardiovascular diseases, Heart failure, Computational biology and bioinformatics, Diseases, Health care

## Abstract

Heart disease is a fatal human disease, rapidly increases globally in both developed and undeveloped countries and consequently, causes death. Normally, in this disease, the heart fails to supply a sufficient amount of blood to other parts of the body in order to accomplish their normal functionalities. Early and on-time diagnosing of this problem is very essential for preventing patients from more damage and saving their lives. Among the conventional invasive-based techniques, angiography is considered to be the most well-known technique for diagnosing heart problems but it has some limitations. On the other hand, the non-invasive based methods, like intelligent learning-based computational techniques are found more upright and effectual for the heart disease diagnosis. Here, an intelligent computational predictive system is introduced for the identification and diagnosis of cardiac disease. In this study, various machine learning classification algorithms are investigated. In order to remove irrelevant and noisy data from extracted feature space, four distinct feature selection algorithms are applied and the results of each feature selection algorithm along with classifiers are analyzed. Several performance metrics namely: accuracy, sensitivity, specificity, AUC, F1-score, MCC, and ROC curve are used to observe the effectiveness and strength of the developed model. The classification rates of the developed system are examined on both full and optimal feature spaces, consequently, the performance of the developed model is boosted in case of high variated optimal feature space. In addition, P-value and Chi-square are also computed for the ET classifier along with each feature selection technique. It is anticipated that the proposed system will be useful and helpful for the physician to diagnose heart disease accurately and effectively.

## Introduction

Heart disease is considered one of the most perilous and life snatching chronic diseases all over the world. In heart disease, normally the heart fails to supply sufficient blood to other parts of the body to accomplish their normal functionality^1^. Heart failure occurs due to blockage and narrowing of coronary arteries. Coronary arteries are responsible for the supply of blood to the heart itself^2^. A recent survey reveals that the United States is the most affected country by heart disease where the ratio of heart disease patients is very high^3^. The most common symptoms of heart disease include physical body weakness, shortness of breath, feet swollen, and weariness with associated signs, etc.^4^. The risk of heart disease may be increased by the lifestyle of a person like smoking, unhealthy diet, high cholesterol level, high blood pressure, deficiency of exercise and fitness, etc.^5^. Heart disease has several types in which coronary artery disease (CAD) is the common one that can lead to chest pain, stroke, and heart attack. The other types of heart disease include heart rhythm problems, congestive heart failure, congenital heart disease (birth time heart disease), and cardiovascular disease (CVD). Initially, traditional investigation techniques were used for the identification of heart disease, however, they were found complex^6^. Owing to the non-availability of medical diagnosing tools and medical experts specifically in undeveloped countries, diagnosis and cure of heart disease are very complex^7^. However, the precise and appropriate diagnosis of heart disease is very imperative to prevent the patient from more damage^8^. Heart disease is a fatal disease that rapidly increases in both economically developed and undeveloped countries. According to a report generated by the World Health Organization (WHO), an average of 17.90 million humans died from CVD in 2016. This amount represents approximately 30% of all global deaths. According to a report, 0.2 million people die from heart disease annually in Pakistan. Every year, the number of victimizing people is rapidly increasing. European Society of Cardiology (ESC) has published a report in which 26.5 million adults were identified having heart disease and 3.8 million were identified each year. About 50–55% of heart disease patients die within the initial 1–3 years, and the cost of heart disease treatment is about 4% of the overall healthcare annual budget^9^.

Conventional invasive-based methods used for the diagnosis of heart disease which were based on the medical history of a patient, physical test results, and investigation of related symptoms by the doctors^10^. Among the conventional methods, angiography is considered one of the most precise technique for the identification of heart problems. Conversely, angiography has some drawbacks like high cost, various side effects, and strong technological knowledge^11^. Conventional methods often lead to imprecise diagnosis and take more time due to human mistakes. In addition, it is a very expensive and computational intensive approach for the diagnosis of disease and takes time in assessment^12^.

To overcome the issues in conventional invasive-based methods for the identification of heart disease, researchers attempted to develop different non-invasive smart healthcare systems based on predictive machine learning techniques namely: Support Vector Machine (SVM), K-Nearest Neighbor (KNN), Naïve Bayes (NB), and Decision Tree (DT), etc.^13^. As a result, the death ratio of heart disease patients has been decreased^14^. In literature, the Cleveland heart disease dataset is extensively utilized by the researchers^15,16^.

In this regard, Robert et al*.*^17^ have used a logistic regression classification algorithm for heart disease detection and obtained an accuracy of 77.1%. Similarly, Wankhade et al*.*^18^ have used a multi-layer perceptron (MLP) classifier for heart disease diagnosis and attained accuracy of 80%. Likewise, Allahverdi et al*.*^19^ have developed a heart disease classification system in which they integrated neural networks with an artificial neural network and attained an accuracy of 82.4%. In a sequel, Awang et al*.*^20^ have used NB and DT for the diagnosis and prediction of heart disease and achieved reasonable results in terms of accuracy. They achieved an accuracy of 82.7% with NB and 80.4% with DT. Oyedodum and Olaniye^21^ have proposed a three-phase system for the prediction of heart disease using ANN. Das and Turkoglu^22^ have proposed an ANN ensemble-based predictive model for the prediction of heart disease. Similarly, Paul and Robin^23^ have used the adaptive fuzzy ensemble method for the prediction of heart disease. Likewise, Tomov et al.^24^ have introduced a deep neural network for heart disease prediction and his proposed model performed well and produced good outcomes. Further, Manogaran and Varatharajan^25^ have introduced the concept of a hybrid recommendation system for diagnosing heart disease and their model has given considerable results. Alizadehsani et al*.*^26^ have developed a non-invasive based model for the prediction of coronary artery disease and showed some good results regarding the accuracy and other performance assessment metrics. Amin et al*.*^27^ have proposed a framework of a hybrid system for the identification of cardiac disease, using machine learning, and attained an accuracy of 86.0%. Similarly, Mohan et al*.*^28^ have proposed another intelligent system that integrates RF with a linear model for the prediction of heart disease and achieved the classification accuracy of 88.7%. Likewise, Liaqat et al*.*^29^ have developed an expert system that uses stacked SVM for the prediction of heart disease and obtained 91.11% classification accuracy on selected features.

The contribution of the current work is to introduce an intelligent medical decision system for the diagnosis of heart disease based on contemporary machine learning algorithms. In this study, 10 different nature of machine learning classification algorithms such as Logistic Regression (LR), Decision Tree (DT), Naïve Bayes (NB), Random Forest (RF), Artificial Neural Network (ANN), etc. are implemented in order to select the best model for timely and accurate detection of heart disease at an early stage. Four feature selection algorithms, Fast Correlation-Based Filter Solution (FCBF), minimal redundancy maximal relevance (mRMR), Least Absolute Shrinkage and Selection Operator (LASSO), and Relief have been used for selecting the vital and more correlated features that have truly reflect the motif of the desired target. Our developed system has been trained and tested on the Cleveland (S_1_) and Hungarian (S_2_) heart disease datasets which are available online on the UCI machine learning repository. All the processing and computations were performed using Anaconda IDE. Python has been used as a tool for implementing all the classifiers. The main packages and libraries used include pandas, NumPy, matplotlib, sci-kit learn (sklearn), and seaborn. The main contribution of our proposed work is given below:

- The performance of all classifiers has been tested on full feature spaces in terms of all performance evaluation matrices specifically accuracy.
- The performances of the classifiers are tested on selected feature spaces, selected through various feature selection algorithms mentioned above.
- The research study recommends that which feature selection algorithm is feasible with which classification algorithm for developing a high-level intelligence system for the diagnosing of heart disease patients.

The rest of the paper is organized as: “Results and discussion” section represents the results and discussion, “Material and methods” section describes the material and methods used in this paper. Finally, we conclude our proposed research work in “Conclusion” section.

## Results and discussion

This section of the paper discusses the experimental results of various contemporary classification algorithms. At first, the performance of all used classification models i.e. K-Nearest Neighbors (KNN), Decision Tree (DT), Extra-Tree Classifier (ETC), Random Forest (RF), Logistic Regression (LR), Naïve Bayes (NB), Artificial Neural Network (ANN), Support Vector Machine (SVM), Adaboost (AB), and Gradient Boosting (GB) along with full feature space is evaluated. After that, four feature selection algorithms (FSA): Fast Correlation-Based Filter (FCBF), Minimal Redundancy Maximal Relevance (mRMR), Least Absolute Shrinkage and Selection Operator (LASSO), and Relief are applied to select the prominent and high variant features from feature space. Furthermore, the selected feature spaces are provided to classification algorithms as input to analyze the significance of feature selection techniques. The cross-validation techniques i.e. k-fold (10-fold) are applied on both the full and selected feature spaces to analyze the generalization power of the proposed model. Various performance evaluation metrics are implemented for measuring the performances of the classification models.

### Classifiers’ predictive outcomes on full feature space

The experimental outcomes of the applied classification algorithms on the full feature space of the two benchmark datasets by using 10-fold cross-validation (CV) techniques are shown in Tables 1 and 2, respectively.

**Table 1:** Classifiers’ success rates on full features using 10-fold CV on S_1_.

Classification model	Accuracy	Sensitivity	Specificity	AUC	Precision	F1-score	MCC
KNN (k = 7)	85.55	85.93	85.17	95.64	86.09	0.86	0.71
DT	86.82	89.73	83.76	91.89	85.40	0.87	0.73
ET	92.09	91.82	92.38	97.92	92.84	0.92	0.84
GB	91.34	90.32	91.52	96.87	92.14	0.92	0.83
RF (n = 100)	89.45	88.57	90.19	94.22	90.82	0.87	0.81
SVM (kernel = ‘rbf’)	84.28	93.15	74.94	92.00	79.77	0.86	0.69
AB	88.09	90.84	84.38	93.92	87.84	0.86	0.76
NB	82.33	86.31	78.15	90.17	80.98	0.83	0.65
LR (C = 10)	84.08	89.92	77.95	92.28	81.24	0.85	0.69
ANN (13, 20, 2)	85.07	84.35	83.72	92.54	83.19	0.82	0.70

**Table 2:** Classifiers’ success rates on full features using 10-fold CV on S_2_.

Classification model	Accuracy	Sensitivity	Specificity	AUC	Precision	F1-score	MCC
KNN (N = 7)	89.16	90.30	87.97	93.30	88.93	0.90	0.78
DT	86.82	89.73	83.76	92.90	85.40	0.87	0.74
ET	96.74	96.36	97.40	98.15	96.10	0.97	0.93
GB	96.05	96.10	97.20	97.85	95.90	0.96	0.92
RF	94.60	94.62	94.19	96.27	94.25	0.94	0.90
SVM (kernel = ‘rbf’)	84.18	92.01	75.95	90.48	80.31	0.86	0.70
AB	92.09	91.82	92.38	97.12	92.84	0.92	0.84
NB	82.33	86.31	78.15	90.71	80.98	0.83	0.65
LR (C = 10)	84.08	89.92	77.95	90.08	81.24	0.85	0.68
ANN (13, 20, 2)	95.80	96.80	95.45	97.70	96.90	0.95	0.91

**Figure 1:**
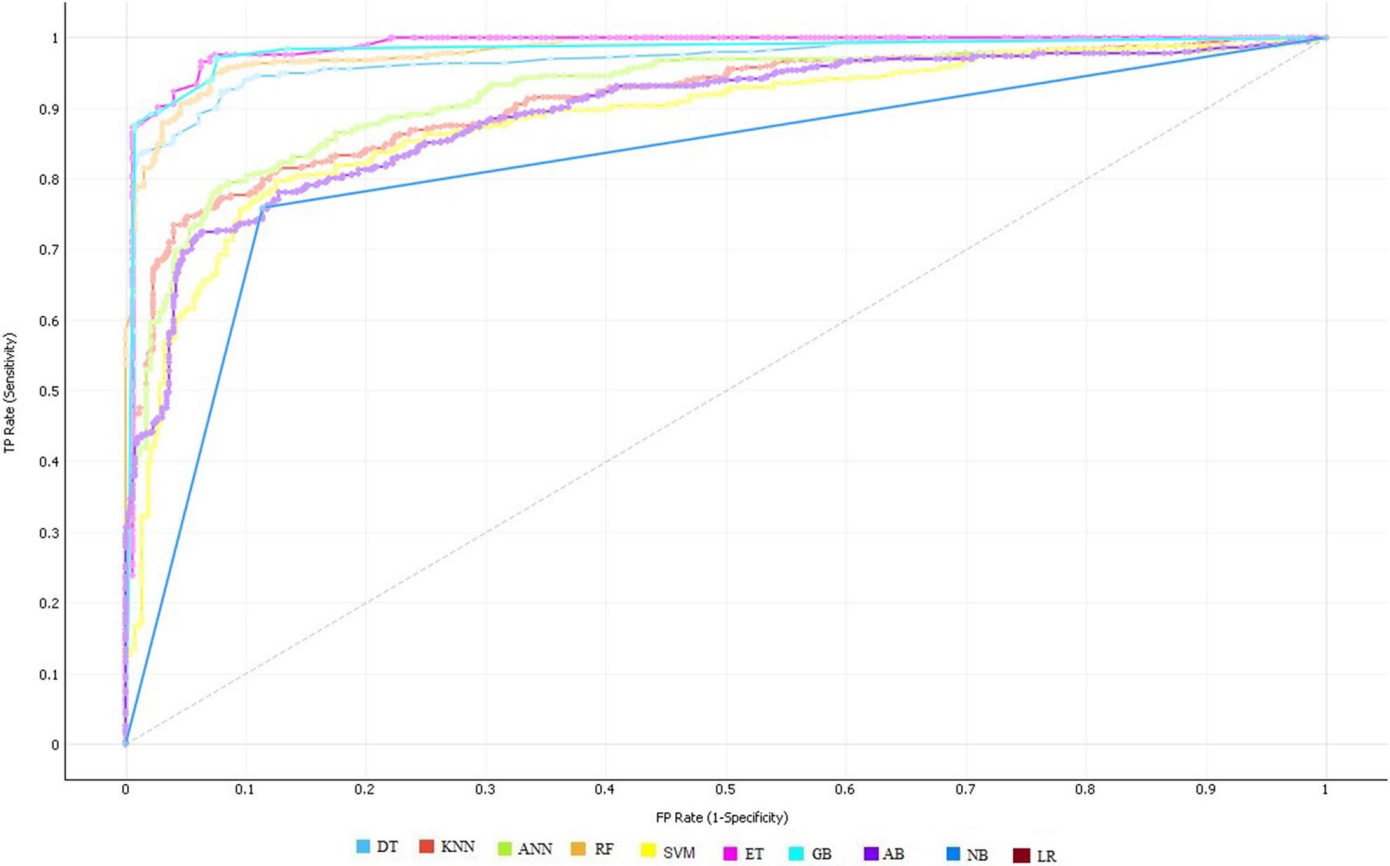
ROC curves of all classifiers on full feature space using 10-fold cross-validation on S_1_.

The experimental results demonstrated that the ET classifier performed quite well in terms of all performance evaluation metrics compared to the other classifiers using 10-fold CV. ET achieved 92.09% accuracy, 91.82% sensitivity, 92.38% specificity, 97.92% AUC, 92.84% Precision, 0.92 F1-Score and 0.84 MCC. The specificity indicates that the diagnosed test was negative and the individual doesn't have the disease. While the sensitivity indicates the diagnostic test was positive and the patient has heart disease. In the case of the KNN classification model, multiple experiments were accomplished by considering various values for k i.e. k = 3, 5, 7, 9, 13, and 15, respectively. Consequently, KNN has shown the best performance at value k = 7 and achieved a classification accuracy of 85.55%, 85.93% sensitivity, 85.17% specificity, 95.64% AUC, 86.09% Precision, 0.86 F1-Score, and 0.71 MCC. Similarly, DT classifier has achieved accuracy of 86.82%, 89.73% sensitivity, 83.76% specificity, 91.89% AUC, 85.40% Precision, 0.87 F1-Score, and 0.73 MCC. Likewise, GB classifier has yielded accuracy of 91.34%, 90.32% sensitivity, 91.52% specificity, 96.87% AUC, 92.14% Precision, 0.92 F1-Score, and 0.83 MCC. After empirically evaluating the success rates of all classifiers, it is observed that ET Classifier out-performed among all the used classification algorithms in terms of accuracy, sensitivity, and specificity. Whereas, NB shows the lowest performance in terms of accuracy, sensitivity, and specificity. The ROC curve of all classification algorithms on full feature space is represented in Fig. 1.

In the case of dataset S_2_, composed of 1025 total instances in which 525 belong to the positive class and 500 instances of having negative class, again ET has obtained quite well results compared to other classifiers using a 10-fold cross-validation test, which are 96.74% accuracy, 96.36 sensitivity, 97.40% specificity, and 0.93 MCC as shown in Table 2.

### Classifiers’ predictive outcomes on selected feature space

#### FCBF feature selection technique

FCBF feature selection technique is applied to select the best subset of feature space. In this attempt, various length of subspaces is generated and tested. Finally, the best results are achieved by classification algorithms on the subset of feature space (n = 6) using a 10-fold CV. Table 3 shows various performance measures of classifiers executed on the selected features space of FCBF.

**Table 3:** Classifiers' performance on optimal feature space of the FCBF feature selection algorithm.

Classification model	Accuracy	Sensitivity	Specificity	AUC	Precision	F1-score	MCC
KNN (K = 7)	87.50	85.55	89.57	92.01	86.25	0.87	0.75
DT	84.97	85.53	89.57	91.49	89.77	0.85	0.70
ET	94.14	94.29	93.98	94.21	94.47	0.94	0.88
GB	93.36	94.67	91.98	93.87	92.44	0.93	0.87
RF	88.48	90.87	85.57	92.42	87.05	0.88	0.77
SVM (kernel = ‘rbf’)	82.62	90.68	74.14	89.68	78.86	0.84	0.66
AB	87.50	89.92	84.96	91.13	86.44	0.88	0.75
NB	81.25	84.22	78.15	88.52	80.55	0.82	0.62
LR (C = 10)	82.33	86.88	77.55	89.51	80.61	0.83	0.65
ANN (13, 20, 2)	88.19	92.20	83.56	90.17	85.48	0.89	0.76

**Figure 2:**
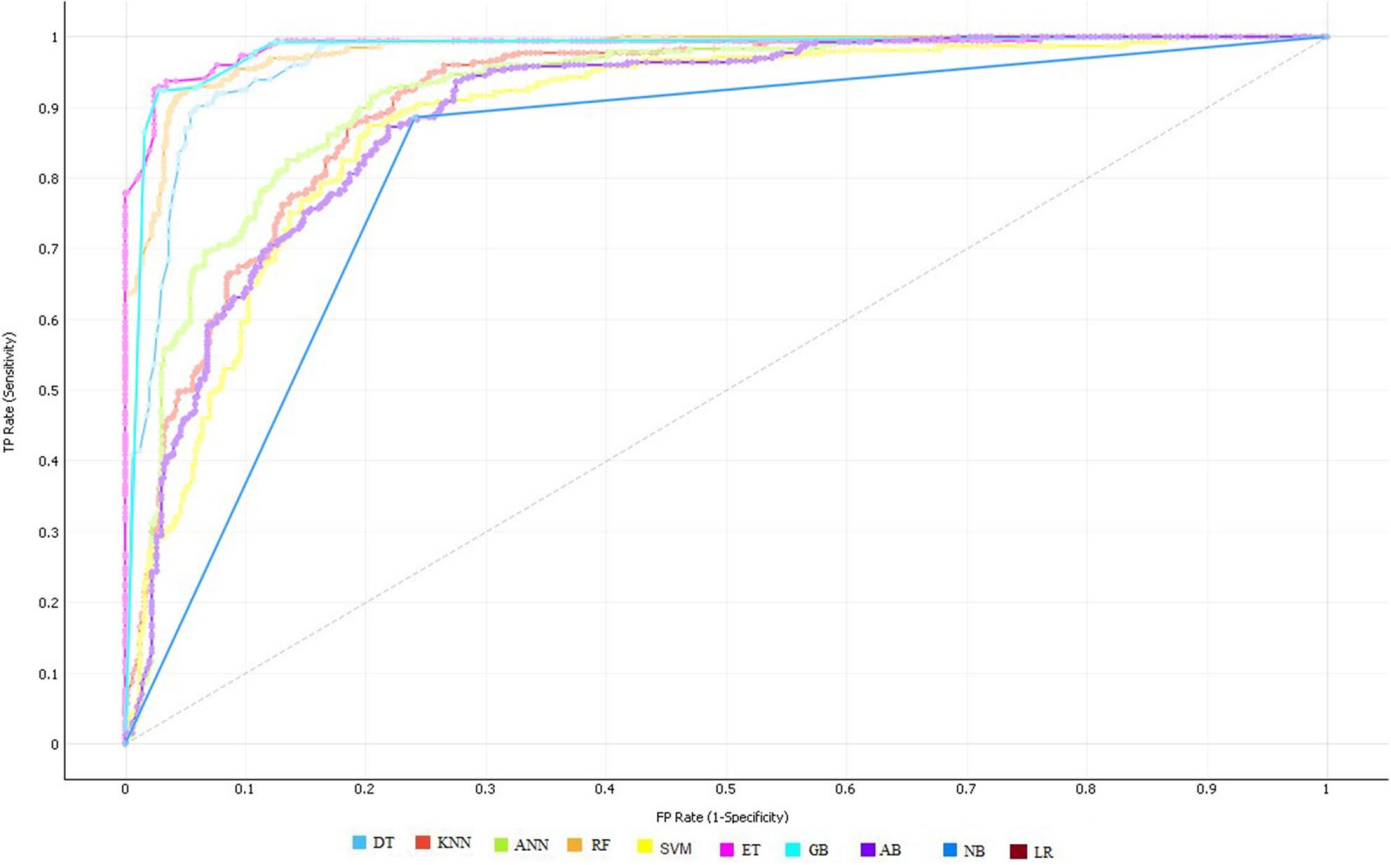
ROC curve of all classifiers on selected features by FCBF feature selection algorithm.

Table 3 demonstrates that the ET classifier obtained quite good results including accuracy of 94.14%, 94.29% sensitivity, and specificity of 93.98%. In contrast, NB reported the lowest performance compared to the other classification algorithms. The performance of classification algorithms is also illustrated in Fig. 2 by using ROC curves.

#### mRMR feature selection technique

mRMR feature selection technique is used in order to select a subset of features that enhance the performance of classifiers. The best results reported on a subset of n = 6 of feature space which is shown in Table 4.

**Table 4:** Classifiers' performance on optimal feature space of the mRMR feature selection algorithm.

Classification model	Accuracy	Sensitivity	Specificity	AUC	Precision	F1-score	MCC
KNN (K = 7)	86.62	85.93	87.37	94.69	87.88	0.87	0.73
DT	82.24	84.98	79.35	87.49	81.38	0.83	0.64
ET	93.42	93.92	93.88	93.23	94.45	0.94	0.88
GB	91.30	92.61	98.92	91.81	90.40	0.91	0.85
RF (n = 100)	88.29	92.20	84.56	94.42	86.33	0.89	0.77
SVM (kernel = ‘rbf’)	82.14	85.36	78.75	86.11	81.11	0.83	0.64
AB	87.41	88.40	86.37	92.81	86.32	0.88	0.75
NB	81.84	81.93	81.76	89.06	82.85	0.82	0.63
LR (C = 10)	82.14	84.03	80.16	86.38	81.81	0.83	0.64
ANN (13, 20, 2)	90.55	91.48	89.58	96.95	90.16	0.90	0.84

**Figure 3:**
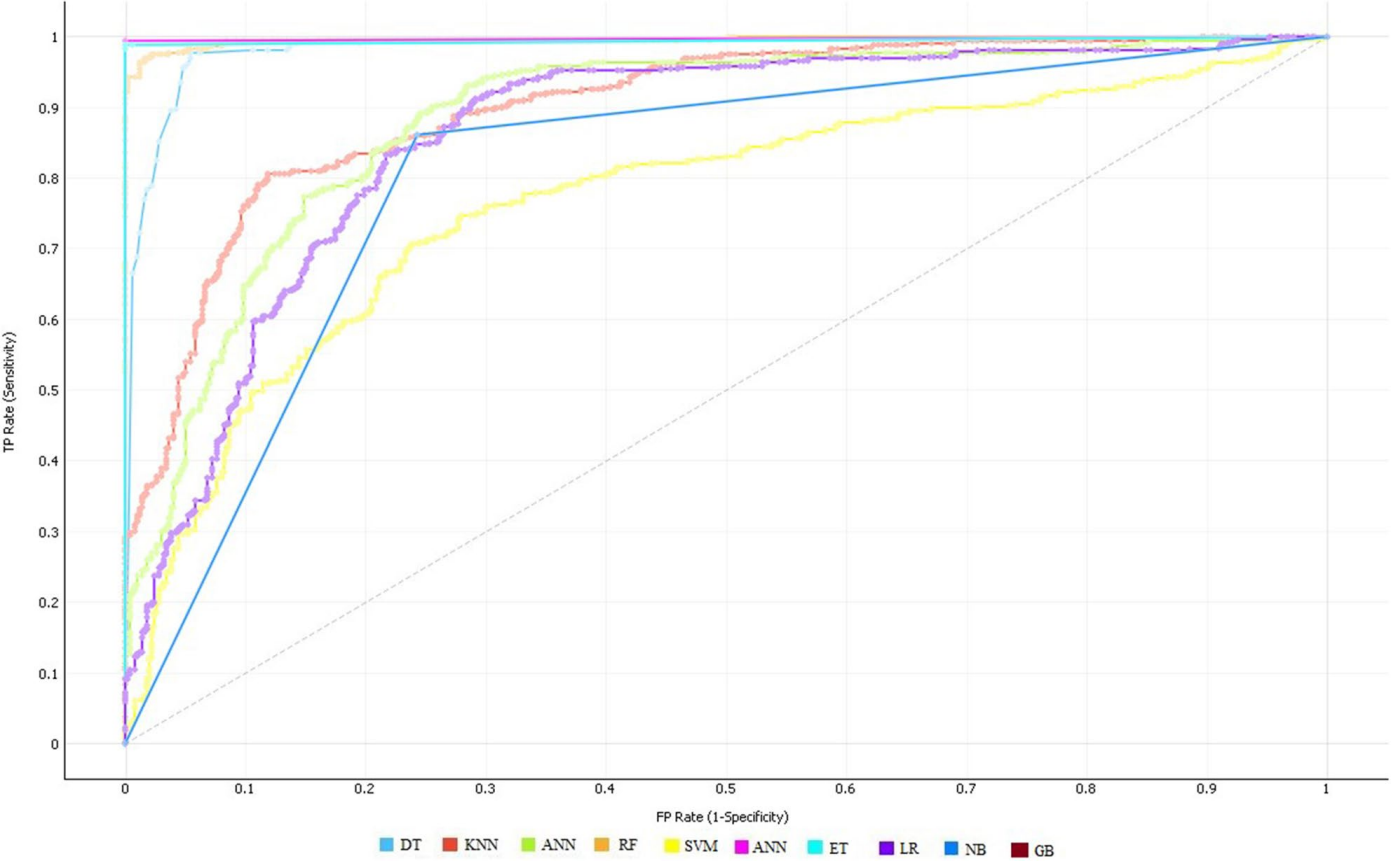
ROC curve of all classifiers on selected features using the mRMR feature selection algorithm.

In the case of mRMR, still, the success rates of the ET classifier are well in terms of all performance evaluation metrics compared to the other classifiers. ET has attained 93.42% accuracy, 93.92% sensitivity, and specificity of 93.88%. In contrast, NB has achieved the lowest outcomes which are 81.84% accuracy. Figure 3 shows the ROC curve of all ten classifiers using the mRMR feature selection algorithm.

#### LASSO feature selection technique

In order to choose the optimal feature space which not only reduces computational cost but also progresses the performance of the classifiers, LASSO feature selection technique is applied. After performing various experiments on different subsets of feature space, the best results are still noted on the subspace of (n = 6). The predicted outcomes of the best-selected feature space are reported in Table 5 using the 10-fold CV.

**Table 5:** Classifiers’ performance on optimal feature space of LASSO feature selection algorithm.

Classification model	Accuracy	Sensitivity	Specificity	AUC	Precision	F1-score	MCC
KNN (K = 7)	85.65	83.84	87.57	92.10	86.82	0.85	0.71
DT	84.28	83.84	84.76	88.68	84.26	0.84	0.68
ET	89.36	88.21	90.58	92.05	88.90	0.88	0.77
GB	88.47	89.54	87.37	92.69	86.39	0.88	0.77
RF	88.18	89.92	85.97	92.52	86.17	0.87	0.76
SVM (kernel = ‘rbf’)	80.57	83.26	77.75	88.03	80.06	0.81	0.61
AB	85.94	86.50	85.37	90.72	84.33	0.86	0.72
NB	82.62	83.84	81.36	86.81	82.76	0.83	0.65
LR (C = 10)	80.77	83.46	77.95	85.00	80.35	0.81	0.61
ANN (13, 20, 2)	87.59	88.02	86.57	92.40	87.58	0.87	0.74

**Figure 4:**
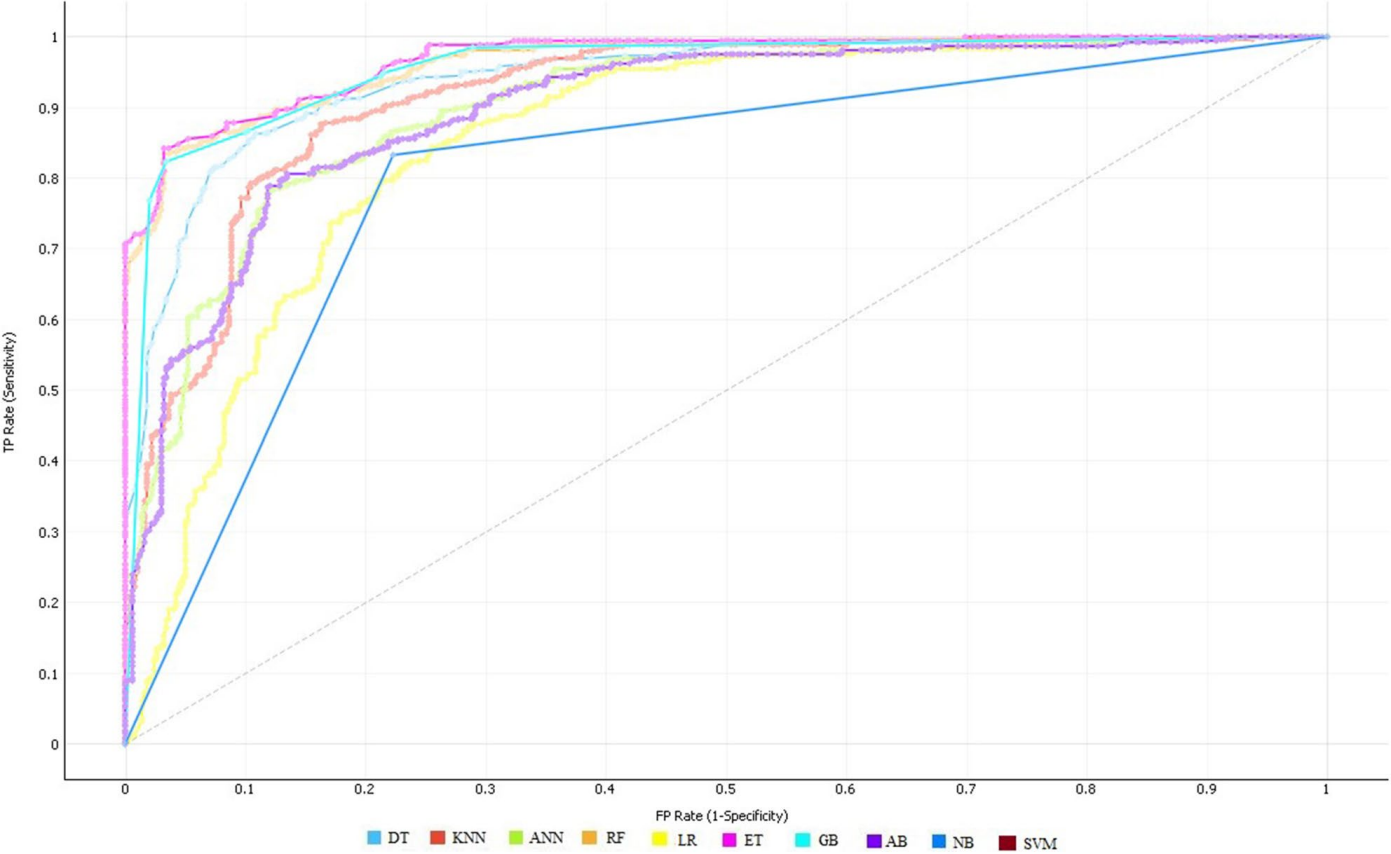
ROC curve of all classifiers on selected feature space using the LASSO feature selection algorithm.

Table 5 demonstrated that the predicted outcomes of the ET classifier are considerable and better compared to the other classifiers. ET has achieved 89.36% accuracy, 88.21% sensitivity, and specificity of 90.58%. Likewise, GB has yielded the second-best result which is the accuracy of 88.47%, 89.54% sensitivity, and specificity of 87.37%. Whereas, LR has performed worse results and achieved 80.77% accuracy, 83.46% sensitivity, and specificity of 77.95%. ROC curves of the classifiers are shown in Fig. 4.

#### Relief feature selection technique

In a sequel, another feature selection technique Relief is applied to investigate the performance of classifiers on different sub-feature spaces by using the wrapper method. After empirically analyzing the results of the classifiers on a different subset of feature spaces, it is observed that the performance of classifiers is outstanding on the sub-space of length (n = 6). The results of the optimal feature space on the 10-fold CV technique are listed in Table 6.

**Table 6:** Classifiers’ performance on optimal feature space of Relief feature selection algorithm.

Classification model	Accuracy	Sensitivity	Specificity	AUC	Precision	F1-score	MCC
KNN (K = 7)	87.11	84.41	89.97	94.08	86.06	0.87	0.74
DT	84.98	89.16	80.56	87.83	82.06	0.85	0.70
ET	94.41	94.93	94.89	94.24	95.46	0.95	0.89
GB	92.35	93.66	98.90	92.86	91.44	0.92	0.87
RF	91.51	92.39	89.37	94.78	89.42	0.91	0.81
SVM (kernel = ‘rbf’)	81.26	87.83	74.34	84.26	82.60	0.82	0.63
AB	83.01	83.26	82.76	88.14	83.78	0.83	0.66
NB	80.29	81.93	78.55	84.94	79.17	0.81	0.60
LR (C = 10)	80.77	84.79	76.55	84.47	78.29	0.82	0.61
ANN (13, 20, 2)	86.72	89.73	83.56	91.79	85.66	0.87	0.73

**Figure 5:**
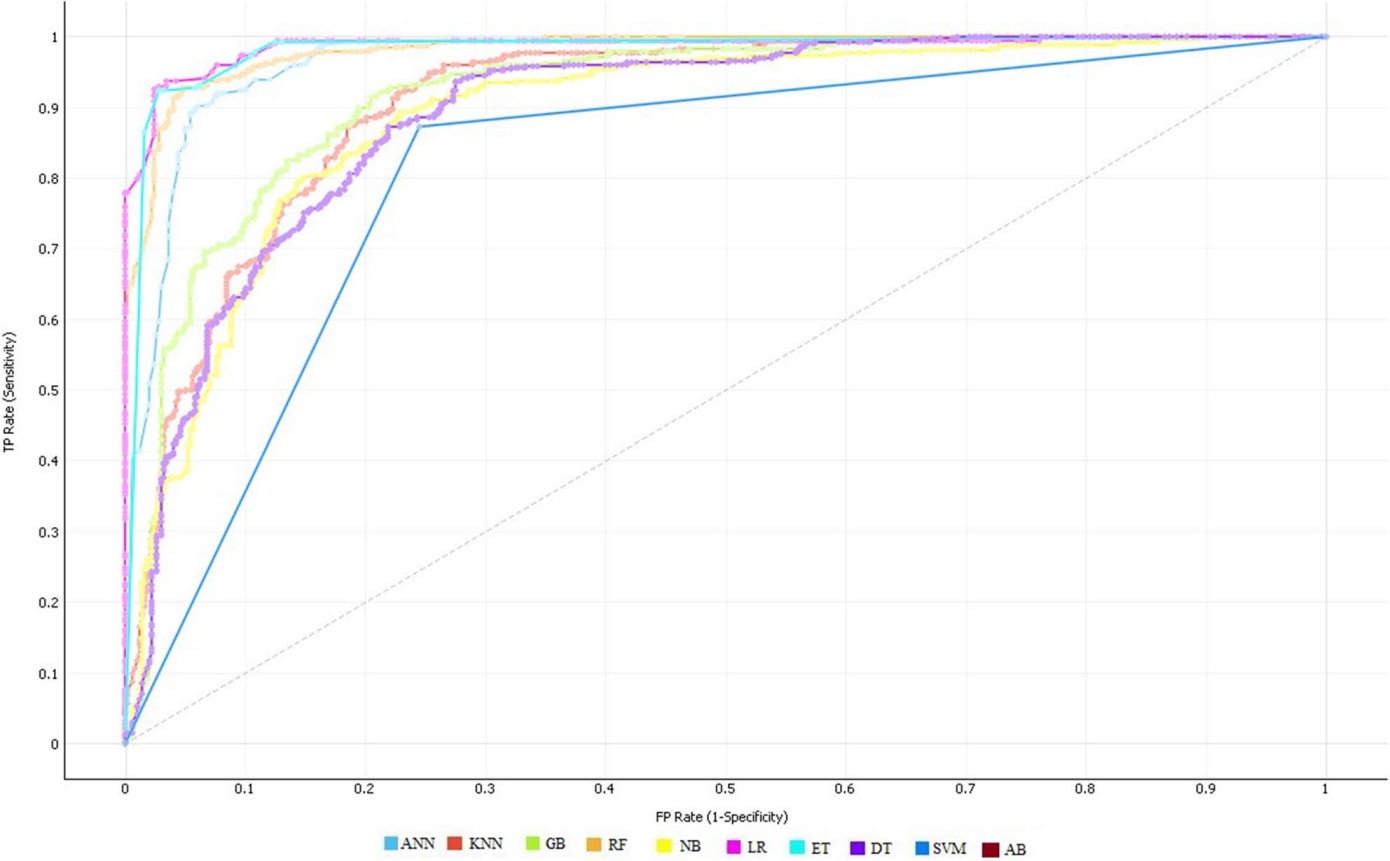
ROC curve of all classifiers on selected features selected by the Relief feature selection algorithm.

Again, the ET classifier performed outstandingly in terms of all performance evaluation metrics as compared to other classifiers. ET has obtained an accuracy of 94.41%, 94.93% sensitivity, and specificity of 94.89%. In contrast, NB has shown the lowest performance and achieved 80.29% accuracy, 81.93% sensitivity, and specificity of 78.55%. The ROC curves of the classifiers are demonstrated in Fig. 5.

After executing classification algorithms along with full and selected feature spaces in order to select the optimal algorithm for the operational engine, the empirical results have revealed that ET performed well not only on all feature space but also on optimal selected feature space among all the used classification algorithms. Furthermore, the ET classifier obtained quite promising accuracy in the case of the Relief feature selection technique which is 94.41%. Overall, the performance of ET is reported better in terms of most of the measures while other classifiers have shown good results in one measure while worse in other measures. In addition, the performance of the ET classifier is also evaluated on a 10-fold CV in combination with different sub-feature spaces of varying length starting from 1 to 12 with a step size of 1 to check the stability and discrimination power of the classifier as described in^30^. Doing so will assist the readers to have a better understanding of the impact, of the number of selected features on the performance of the classifiers. The same process is repeated for another dataset i.e. S_2_ (Hungarian heart disease dataset) as well, to know the impact of selected features on the classification performance.

Tables 7 and 8 shows the performance of the ET classifier using 10-fold CV in combination with different feature sub-spaces starting from 1 to 12 with a step size of 1. The experimental results show that the performance of the ET classifier is affected significantly by using the varying length of sub-feature spaces. Finally, it is concluded that all these achievements are ascribed with the best selection of Relief feature selection technique which not only reduces the feature space but also enhances the predictive power of classifiers. In addition, the ET classifier has also played a quite promising role in these achievements because it has clearly and precisely learned the motif of the target class and reflected it truly. In addition, the performance of the ET classifier is also evaluated on 5-fold and 7-fold CV in combination with different sub-spaces of length 5 and 7 to check the stability and discrimination power of the classifier. It is also tested on another dataset S_2_ (Hungarian heart disease dataset). The results are shown in supplementary materials.

**Table 7:** Performance of ET classifier using 10-fold CV on different sub-feature spaces on S_1_.

Features size	Accuracy	Sensitivity	Specificity	AUC	Precision	F1-score	MCC
1	72.86	73.33	73.91	77.02	75.71	0.75	0.47
2	75.81	76.36	75.36	75.19	78.70	0.77	0.52
3	81.12	85.45	76.08	88.13	83.38	0.84	0.62
4	85.11	86.84	80.26	90.56	84.83	0.85	0.68
5	92.60	91.65	92.72	95.80	91.48	0.93	0.85
6	94.41	94.93	94.89	94.24	95.46	0.95	0.89
7	92.12	91.85	92.42	96.98	92.86	0.92	0.84
8	92.12	91.85	92.42	96.98	92.86	0.92	0.84
9	92.10	91.84	92.39	96.94	92.84	0.92	0.84
10	92.10	91.84	92.39	96.94	92.84	0.92	0.84
11	92.09	91.82	92.38	97.92	92.84	0.92	0.84
12	92.09	91.82	92.38	97.92	92.84	0.92	0.84

**Table 8:** Performance of ET classifier using 10-fold CV on different sub-feature spaces on S_2_.

Features size	Accuracy	Sensitivity	Specificity	AUC	Precision	F1-score	MCC
1	75.98	76.80	75.15	077.11	76.69	0.76	0.52
2	82.62	76.23	89.37	91.48	88.43	0.82	0.66
3	83.80	79.27	88.57	93.27	88.19	0.83	0.68
4	91.99	90.30	93.78	98.25	93.91	0.92	0.86
5	96.80	97.20	96.25	97.80	97.12	0.97	0.94
6	98.04	97.21	98.15	98.95	98.03	0.98	0.95
7	96.81	97.24	96.30	98.16	97.12	0.97	0.94
8	96.80	97.22	96.28	98.14	97.12	0.97	0.94
9	96.80	97.22	96.28	98.14	97.12	0.97	0.94
10	96.78	97.16	96.34	97.96	96.94	0.97	0.94
11	96.76	97.22	96.24	97.82	96.88	0.97	0.94
12	96.74	96.36	97.40	98.15	96.10	0.97	0.93

In Table 9, P-value and Chi-Square values are also computed for the ET classifier in combination with the optimal feature spaces of different feature selection techniques.

**Table 9:** P-value and chi-square of ET classifier on different feature selection techniques.

Classifier + feature selection	Chi-square	P-value (α = 0.05)
ET + relief	116.7555	< 0.00001
ET + FCBF	114.5667	< 0.00001
ET + mRMR	104.2308	< 0.00001
ET + LASSO	88.1475	< 0.00001

### Performance comparison with existing models

Further, a comparative study of the developed system is conducted with other states of the art machine learning approaches discussed in the literature. Table 10 represents, a brief description and classification accuracies of those approaches. The results demonstrate that our proposed model success rate is high compared to existing models in the literature.

**Table 10:** Classification accuracy of the developed system and other approaches in the literature using heart disease dataset.

Publications	Approach	Accuracy
Amin et al.^27^	Hybrid framework	86.00
Mohan et al.^28^	HRFLM	88.70
Kumar et al.^36^	ANFIS	91.00
Samuel et al.^10^	ANN-fuzzy-AHP	91.10
Liaqat et al.^29^	Stacked SVM	91.11
Developed model	Intelligent framework (full features)	92.09
Developed model	Intelligent framework (selected-features)	94.41

## Material and methods

The subsections represent the materials and the methods that are used in this paper.

### Dataset

The first and rudimentary step of developing an intelligent computational model is to construct or develop a problem-related dataset that truly and effectively reflects the pattern of the target class. Well organized and problem-related dataset has a high influence on the performance of the computational model. Looking at the significance of the dataset, two datasets i.e. the Cleveland heart disease dataset S_1_ and Hungarian heart disease dataset (S_2_) are used, which are available online at the University of California Irvine (UCI) machine learning repository and UCI Kaggle repository, and various researchers have used it for conducting their research studies^28,31,32^. The S1 consists of 304 instances, where each instance has distinct 13 attributes along with the target labels and are selected for training. The dataset is composed of two classes, presence or absence of heart disease. The S_2_ is composed of 1025 instances in which 525 instances belong to positive class while the rest of 500 instances have negative class. The description of attributes of both the datasets is the same, and both have similar attributes. The complete description and information of the datasets with 13 attributes are given in Table 11.

**Table 11:** Description of CHDD and HHDD datasets.

S. no	Feature name	Feature code	Description	Range of values
1	Age	Age	Age in years	26 < age < 88
2	Sex	Sex	Male = 1	1
			Female = 0	0
3	Chest pain type	CPT	Atypical angina	0
			Typical angina	1
			Asymptotic	2
			Non-anginal pain	3
4	Resting blood pressure	RBP	mmHg in the hospital	94–200
5	Serum cholesterol	SCH	in mg/dl	120–564
6	Fasting blood sugar	FBS	FBS > 120 mg/dl (0 = false, 1 = true)	0 1
7	Resting electrocardiographic results	RECG	0 = normal	0
			1 = having ST-T	1
			2 = Hypertrophy	2
8	Thallium scan	THA	0 = normal	0
			1 = fixed defect	1
			2 = reversible defect	2
9	Number of major vessels colored by fluoroscopy	VCA	–	0 1 2 3
10	The slope of peak exercise ST segments	PES	0 = up sloping	0
			1 = flat/ no slope	1
			2 = down sloping	2
11	Old peak	OPK	–	0–6.5
12	Exercise-induced angina	EIA	0 = no	0
			1 = yes	1
13	Maximum heart rate	MHR	–	70–204

### Proposed system methodology

**Figure 6:**
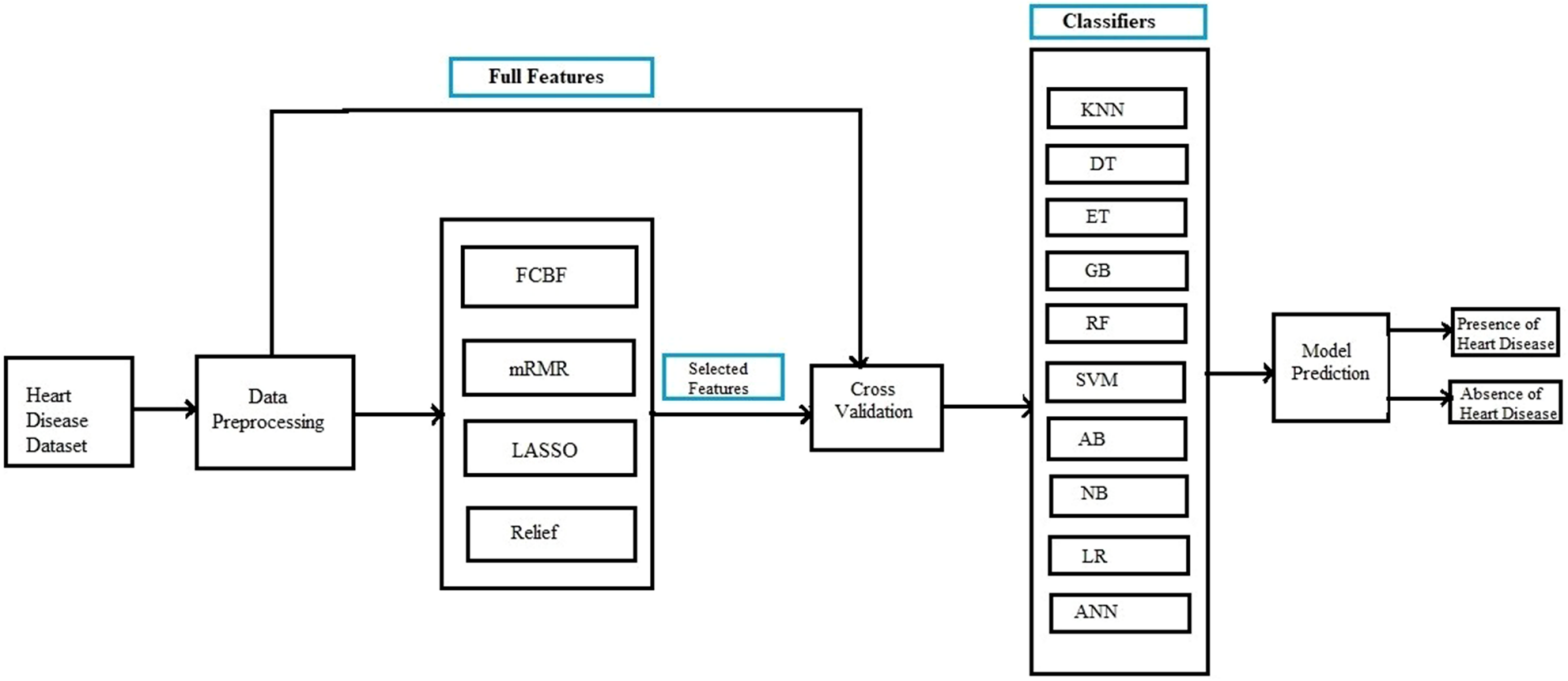
An Intelligent Hybrid Framework for the prediction of heart disease.

The main theme of the developed system is to identify heart problems in human beings. In this study, four distant feature selection techniques namely: FCBF, mRMR, Relief, and LASSO are applied on the provided dataset in order to remove noisy, redundant features and select variant features, consequently may cause of enhancing the performance of the proposed model. Various machine learning classification algorithms are used in this study which include, KNN, DT, ETC, RF, LR, NB, ANN, SVM, AB, and GB. Different evaluation metrics are computed to assess the performance of classification algorithms. The methodology of the proposed system is carried out in five stages which include dataset preprocessing, selection of features, cross-validation technique, classification algorithms, and performance evaluation of classifiers. The framework of the proposed system is illustrated in Fig. 6.

#### Preprocessing of data

Data preprocessing is the process of transforming raw data into meaningful patterns. It is very crucial for a good representation of data. Various preprocessing approaches such as missing values removal, standard scalar, and Min–Max scalar are used on the dataset in order to make it more effective for classification.

#### Feature selection algorithms

**Figure 7:**
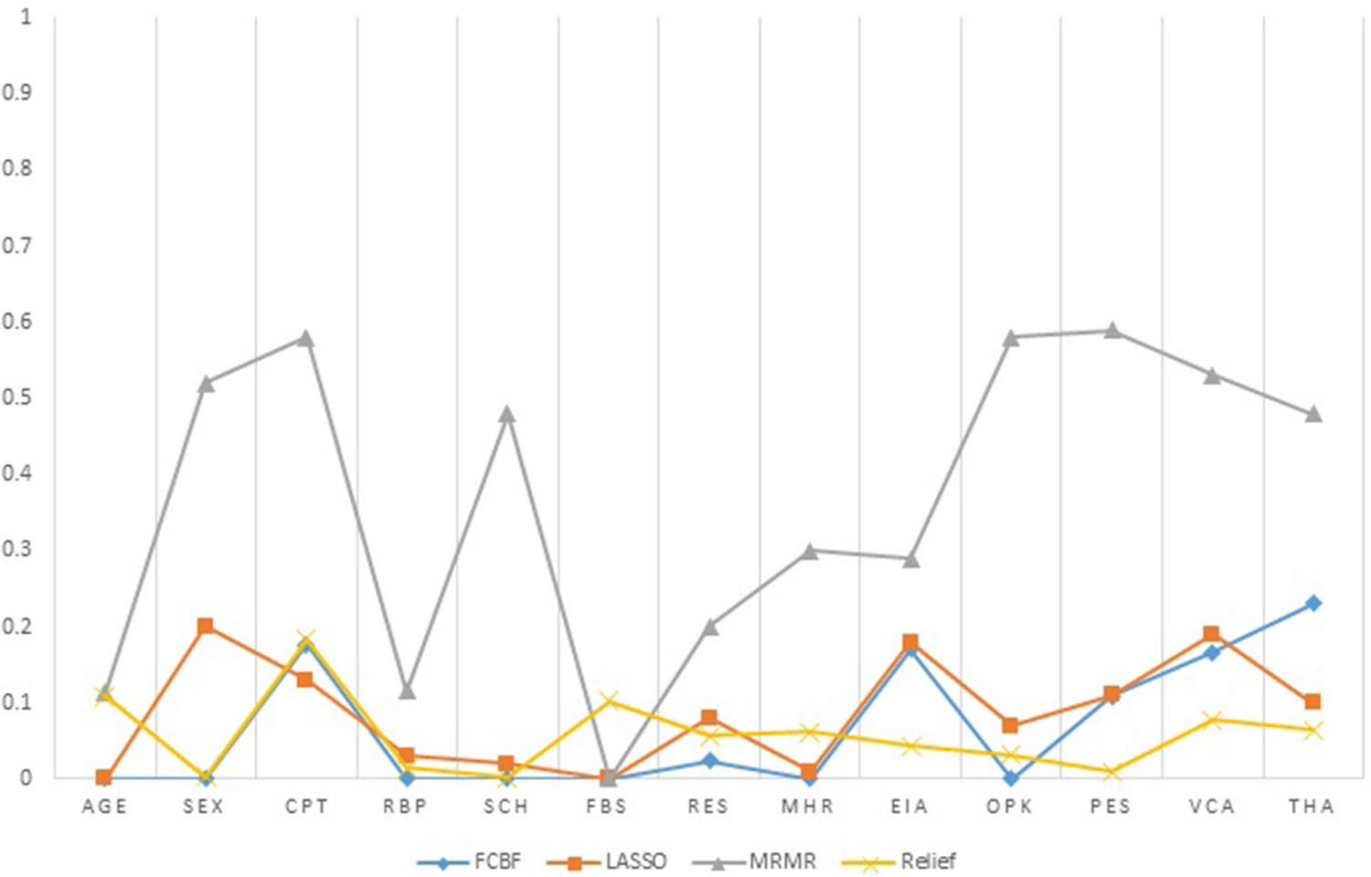
Features ranking by four feature selection algorithms (FCBF, LASSO, mRMR, Relief).

Feature selection technique selects the optimal features sub-space among all the features in a dataset. It is very crucial because sometimes, the classification performance degrades due to irrelevant features in the dataset. The feature selection technique improves the performance of classification algorithms and also reduces their execution time. In this research study, four feature selection techniques are used and are listed below:

- *Fast correlation-based filter (FCBF):* FCBF feature selection algorithm follows a sequential search strategy. It first selects full features and then uses symmetric uncertainty for measuring the dependencies of the features on each other and how they affect the target output label. After this, it selects the most important features using the backward sequential search strategy. FCBF outperforms on high dimensional datasets. Table 12 shows the results of the selected features (n = 6) by using the FCBF feature selection algorithm. Each attribute is given a weight based on its importance. According to the FCBF feature selection technique, the most important features are THA and CPT as shown in Table 12. The ranking that the FCBF gives to all the features of the dataset is shown in Fig. 7.
- *Minimal redundancy maximal relevance (mRMR):* mRMR uses the heuristic approach for selecting the most vital features that have minimum redundancy and maximum relevance. It selects those features which are useful and relevant to the target. As it follows a heuristic approach so, it checks one feature at a time and then computes its pairwise redundancy with the other features. The mRMR feature selection algorithm is not suitable for high domain feature problems^33^. The results of selected features by the mRMR feature selection algorithm (n = 6) are listed in Table 13. In addition, among these attributes, PES and CPT have the highest score. Figure 7 describes the attributes ranking given by the mRMR feature selection algorithm to all attributes in the feature space.

**Table 12:** Selected Features by FCBF algorithm and their Scores.

S. no.	Features	Feature codes	Score
1	8	THA	0.230
2	3	CPT	0.177
3	12	EIA	0.171
4	9	VCA	0.166
5	10	PES	0.109
6	7	RES	0.024

**Table 13:** Selected features by mRMR algorithm and their scores.

S. no.	Features	Feature codes	Score
1	3	PES	0.59
2	5	CPT	0.58
3	10	OPK	0.57
4	9	VCA	0.53
5	2	SEX	0.52
6	8	THA	0.48
- *Least absolute shrinkage and selection operator (LASSO)* LASSO selects features based on updating the absolute value of the features coefficient. In updating the features coefficient values, zero becoming values are removed from the features subset. LASSO outperforms with low feature coefficient values. The features having high coefficient values will be selected in the subset of features and the rest will be eliminated. Moreover, some irrelevant features with higher coefficient values may be selected and are included in the subset of features^30^. Table 14 represents the six most profound attributes which have a great correlation with the target and their scores selected by the LASSO feature selection algorithm. Figure 7 represents the important features and their scoring values given by the LASSO feature selection algorithm.

**Table 14:** Selected features by LASSO algorithm and their scores.

S. no..	Features	Feature codes	Score
1	2	SEX	0.20
2	9	VCA	0.19
3	12	EIA	0.18
4	3	CPT	0.13
5	10	PES	0.11
6	8	THA	0.10
- *Relief feature selection algorithm* Relief utilizes the concept of instance-based learning which allocates weight to each attribute based on its significance. The weight of each attribute demonstrates its capability to differentiate among class values. Attributes are rated by weights, and those attributes whose weight is exceeding a user-specified cutoff, are chosen as the final subset^34^ . The relief feature selection algorithm selects the most significant attributes which have more effect on the target^35^ . The algorithm operates by selecting instances randomly from the training samples. The nearest instance of the same class (nearest hit) and opposite class (nearest miss) is identified for each sampled instance. The weight of an attribute is updated according to how well its values differentiate between the sampled instance and its nearest miss and hit. If an attribute discriminates amongst instances from different classes and has the same value for instances of the same class, it will get a high weight.

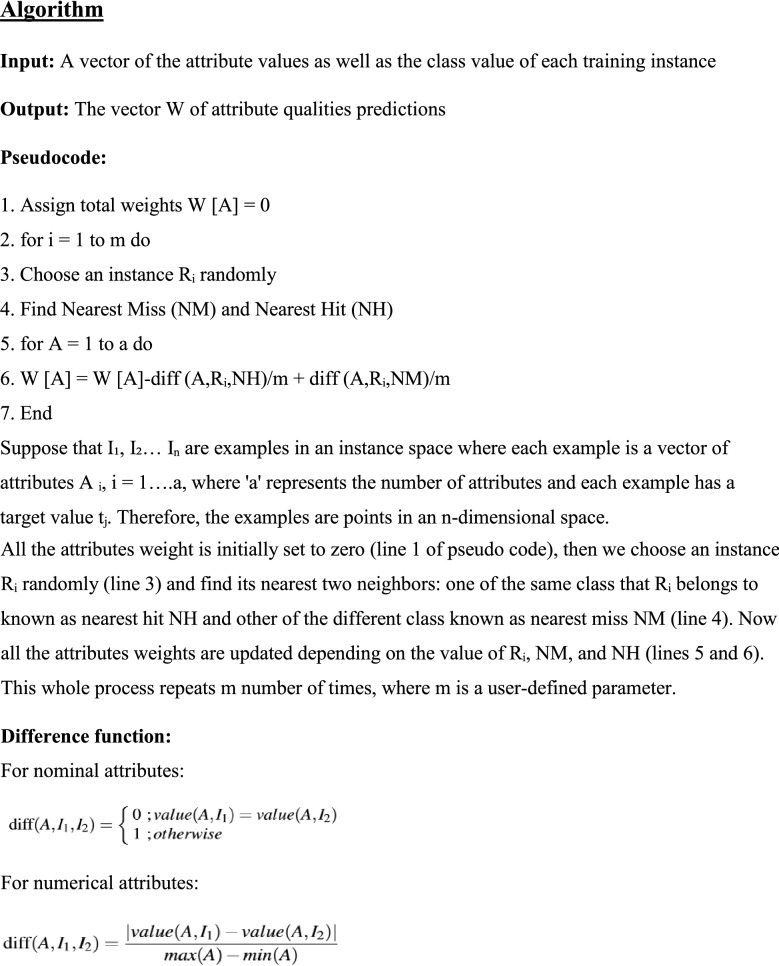


The weight updating of attributes works on a simple idea (line 6). That if instance R_i_ and NH have dissimilar value (i.e. the diff value is large), that means the attribute splits two instances with the same class which is not worthwhile, and thus we reduce the attributes weight. On the other hand, if the instance R_i_ and NM have a distinct value that means the attribute separates the two instances with a different class, which is desirable. The six most important features selected by the Relief algorithm are listed in descending order in Table 15. Based on weight values the most vital features are CPT and Age. Figure 7 demonstrates the important features and their ranking given by the Relief feature selection algorithm.

**Table 15:** Selected features by relief algorithm and their scores.

S. no.	Features	Feature codes	Score
1	3	CPT	0.184
2	1	Age	0.109
3	6	FBS	0.102
4	9	VCA	0.078
5	8	THA	0.064
6	13	MHR	0.062

#### Machine learning classification algorithms

Various machine learning classification algorithms are investigated for early detection of heart disease, in this study. Each classification algorithm has its significance and the importance is reported varied from application to application. In this paper, 10 distant nature of classification algorithms namely: KNN, DT, ET, GB, RF, SVM, AB, NB, LR, and ANN are applied to select the best and generalize prediction model.

#### Classifier validation method

Validation of the prediction model is an essential step in machine learning processes. In this paper, the K-Fold cross-validation method is applied to validating the results of the above-mentioned classification models.

##### K-fold cross validation (CV)

In K-Fold CV, the whole dataset is split into k equal parts. The (k-1) parts are utilized for training and the rest is used for the testing at each iteration. This process continues for k-iteration. Various researchers have used different values of k for CV. Here k = 10 is used for experimental work because it produces good results. In tenfold CV, 90% of data is utilized for training the model and the remaining 10% of data is used for the testing of the model at each iteration. At last, the mean of the results of each step is taken which is the final result.

#### Performance evaluation metrics

For measuring the performance of the classification algorithms used in this paper, various evaluation matrices have been implemented including accuracy, sensitivity, specificity, f1-score, recall, Mathew Correlation-coefficient (MCC), AUC-score, and ROC curve. All these measures are calculated from the confusion matrix described in Table 16.

**Table 16:** Confusion matrix.

	Predicted (no disease)	Predicted (heart disease)
Actual (no disease)	TN	FP
Actual (heart disease)	FN	TP

In confusion matrix True Negative (TN) shows that the patient has not heart disease and the model also predicts the same i.e. a healthy person is correctly classified by the model.

True Positive (TP) represents that the patient has heart disease and the model also predicts the same result i.e. a person having heart disease is correctly classified by the model.

False Positive (FP) demonstrates that the patient has not heart disease but the model predicted that the patient has i.e. a healthy person is incorrectly classified by the model. This is also called a type-1 error.

False Negative (FN) notifies that the patient has heart disease but the model predicted that the patient has not i.e. a person having heart disease is incorrectly classified by the model. This is also called a type-2 error.

*Accuracy* Accuracy of the classification model shows the overall performance of the model and can be calculated by the formula given below:

$${\text{Accuracy}} = \frac{{{\text{TP}} + {\text{TN}}}}{{{\text{TP}} + {\text{TN}} + {\text{FP}} + {\text{FN}}}}*100$$

*Specificity* specificity is a ratio of the recently classified healthy people to the total number of healthy people. It means the prediction is negative and the person is healthy. The formula for calculating specificity is given as follows:

$${\text{Specificity}} = \frac{{{\text{TN}}}}{{{\text{TN}} + {\text{FP}}}}*100$$

*Sensitivity* Sensitivity is the ratio of recently classified heart patients to the total patients having heart disease. It means the model prediction is positive and the person has heart disease. The formula for calculating sensitivity is given below:

$${\text{Sensitivity}} = \frac{{{\text{TP}}}}{{{\text{TP}} + {\text{FN}}}}*100$$

Precision: Precision is the ratio of the actual positive score and the positive score predicted by the classification model/algorithm. Precision can be calculated by the following formula:

$$\text{Precision}=\frac{\text{TP}}{\text{TP}+\text{FP}}*100$$

*F1-score* F1 is the weighted measure of both recall precision and sensitivity. Its value ranges between 0 and 1. If its value is one then it means the good performance of the classification algorithm and if its value is 0 then it means the bad performance of the classification algorithm.

$${\text{F}}1 = \frac{{2*\left( {{\text{Precision}}*{\text{Recall}}} \right)}}{{{\text{Precision}} + {\text{Recall}}}}$$

*MCC* It is a correlation coefficient between the actual and predicted results. MCC gives resulting values between − 1 and + 1. Where − 1 represents the completely wrong prediction of the classifier.0 means that the classifier generates random prediction and + 1 represents the ideal prediction of the classification models. The formula for calculating MCC values is given below:

$${\text{MCC}} = \frac{{{\text{TP}}*{\text{TN}} - {\text{FP}}*{\text{FN}}}}{{\sqrt {\left( {{\text{TP}} + {\text{FP}}} \right)\left( {{\text{TP}} + {\text{FN}}} \right)\left( {{\text{TN}} + {\text{FP}}} \right)\left( {{\text{TN}} + {\text{FN}}} \right)} }}$$

Finally, we will examine the predictability of the machine learning classification algorithms with the help of the receiver optimistic curve (ROC) which represents a graphical demonstration of the performance of ML classifiers. The area under the curve (AUC) describes the ROC of a classifier and the performance of the classification algorithms is directly linked with AUC i.e. larger the value of AUC greater will be the performance of the classification algorithm.

In this study, 10 different machine learning classification algorithms namely: LR, DT, NB, RF, ANN, KNN, GB, SVM, AB, and ET are implemented in order to select the best model for early and accurate detection of heart disease. Four feature selection algorithms such as FCBF, mRMR, LASSO, and Relief have been used to select the most vital and correlated features that truly reflect the motif of the desired target. Our developed intelligent computational model has been trained and tested on two datasets i.e. Cleveland (S1) and Hungarian (S2) heart disease datasets. Python has been used as a tool for implementation and simulating the results of all the utilized classification algorithms.

The performance of all classification models has been tested in terms of various performance metrics on full feature space as well as selected feature spaces, selected through various feature selection algorithms. This research study recommends that which feature selection algorithm is feasible with which classification model for developing a high-level intelligent system for the diagnosis of a patient having heart disease. From simulation results, it is observed that ET is the best classifier while relief is the optimal feature selection algorithm. In addition, P-value and Chi-square are also computed for the ET classifier along with each feature selection algorithm. It is anticipated that the proposed system will be useful and helpful for the doctors and other care-givers to diagnose a patient having heart disease accurately and effectively at the early stages.

## Conclusion

Heart disease is one of the most devastating and fatal chronic diseases that rapidly increase in both economically developed and undeveloped countries and causes death. This damage can be reduced considerably if the patient is diagnosed in the early stages and proper treatment is provided to her. In this paper, we developed an intelligent predictive system based on contemporary machine learning algorithms for the prediction and diagnosis of heart disease. The developed system was checked on two datasets i.e. Cleveland (S1) and Hungarian (S2) heart disease datasets. The developed system was trained and tested on full features and optimal features as well. Ten classification algorithms including, KNN, DT, RF, NB, SVM, AB, ET, GB, LR, and ANN, and four feature selection algorithms such as FCBF, mRMR, LASSO, and Relief are used. The feature selection algorithm selects the most significant features from the feature space, which not only reduces the classification errors but also shrink the feature space. To assess the performance of classification algorithms various performance evaluation metrics were used such as accuracy, sensitivity, specificity, AUC, F1-score, MCC, and ROC curve. The classification accuracies of the top two classification algorithms i.e. ET and GB on full features were 92.09% and 91.34% respectively. After applying feature selection algorithms, the classification accuracy of ET with the relief feature selection algorithm increases from 92.09 to 94.41%. The accuracy of GB increases from 91.34 to 93.36% with the FCBF feature selection algorithm. So, the ET classifier with the relief feature selection algorithm performs excellently. P-value and Chi-square are also computed for the ET classifier with each feature selection technique. The future work of this research study is to use more optimization techniques, feature selection algorithms, and classification algorithms to improve the performance of the predictive system for the diagnosis of heart disease.

## Supplementary information

Supplementary Information.
