# Supplementary material for: Early and accurate detection and diagnosis of heart disease using intelligent computational model
Source: Sci Rep. 2020 Nov 12;10:19747. doi: 10.1038/s41598-020-76635-9 (PMC7665174; doi:10.1038/s41598-020-76635-9)
Supplement: Supplementary file 1 — Supplementary Information. [file 41598_2020_76635_MOESM1_ESM.docx]

Supplementary materials

Table 1: Classifiers’ success rates on full features using 7-fold CV on S1

| Classification Model | Accuracy | Sensitivity | Specificity | AUC | Precision | F1-score | MCC |
| --- | --- | --- | --- | --- | --- | --- | --- |
| KNN (k=7) | 84.17 | 85.90 | 84.11 | 95.39 | 86.05 | 0.85 | 0.70 |
| DT | 86.80 | 89.13 | 83.68 | 90.69 | 85.80 | 0.87 | 0.73 |
| ET | 91.29 | 91.62 | 92.04 | 96.12 | 91.96 | 0.92 | 0.83 |
| GB | 91.14 | 89.21 | 92.12 | 95.84 | 91.24 | 0.92 | 0.82 |
| RF (n=100) | 89.45 | 88.67 | 90.29 | 94.24 | 89.06 | 0.87 | 0.80 |
| SVM (kernel=’rbf’) | 82.60 | 91.06 | 73.64 | 90.00 | 82.27 | 0.84 | 0.67 |
| AB | 87.20 | 90.14 | 85.16 | 92.90 | 87.94 | 0.86 | 0.75 |
| NB | 82.20 | 86.22 | 78.12 | 90.13 | 80.86 | 0.82 | 0.65 |
| LR (C=10) | 84.18 | 89.90 | 78.04 | 91.38 | 84.14 | 0.84 | 0.69 |
| ANN (13, 20, 2) | 84.97 | 84.30 | 83.78 | 92.58 | 83.10 | 0.83 | 0.71 |

Table 2: Classifiers’ success rates on full features using 5-fold CV on S1

| Classification Model | Accuracy | Sensitivity | Specificity | AUC | Precision | F1-score | MCC |
| --- | --- | --- | --- | --- | --- | --- | --- |
| KNN (k=7) | 82.90 | 84.86 | 83.88 | 94.23 | 85.13 | 0.84 | 0.70 |
| DT | 86.60 | 89.05 | 83.54 | 90.12 | 86.74 | 0.86 | 0.73 |
| ET | 91.28 | 91.60 | 92.10 | 96.10 | 91.82 | 0.92 | 0.83 |
| GB | 91.04 | 89.16 | 92.06 | 95.80 | 91.12 | 0.92 | 0.82 |
| RF (n=100) | 89.40 | 88.64 | 90.22 | 94.16 | 89.16 | 0.88 | 0.80 |
| SVM (kernel=’rbf’) | 82.48 | 91.00 | 73.60 | 90.00 | 82.34 | 0.83 | 0.67 |
| AB | 87.20 | 90.24 | 85.06 | 92.86 | 87.64 | 0.87 | 0.75 |
| NB | 82.10 | 86.16 | 78.18 | 90.05 | 80.36 | 0.81 | 0.65 |
| LR (C=10) | 84.28 | 89.88 | 78.14 | 91.30 | 84.24 | 0.85 | 0.70 |
| ANN (13, 20, 2) | 85.06 | 84.42 | 83.84 | 92.64 | 84.08 | 0.84 | 0.72 |

Table 3. Performance of ET classifier on different CV and subset feature spaces on S1

| Selected features (n=7) of Cleveland heart disease dataset | | | | | | | |
| --- | --- | --- | --- | --- | --- | --- | --- |
| **K-Fold CV** | **Accuracy** | **Sensitivity** | **Specificity** | **AUC** | **Precision** | **F1-score** | **MCC** |
| 10-fold | 92.12 | 91.85 | 92.42 | 96.98 | 92.86 | 0.92 | 0.84 |
| 7-fold | 91.39 | 91.68 | 92.14 | 96.14 | 92.08 | 0.93 | 0.83 |
| 5-fold | 91.31 | 91.63 | 92.16 | 96.02 | 91.42 | 0.93 | 0.83 |
| Selected features (n=6) of Cleveland heart disease dataset | | | | | | | |
| 10-fold | 94.41 | 94.93 | 94.89 | 94.24 | 95.46 | 0.95 | 0.89 |
| 7-fold | 94.10 | 93.96 | 94.80 | 94.12 | 94.24 | 0.94 | 0.89 |
| 5-fold | 93.26 | 91.70 | 92.62 | 93.81 | 93.92 | 0.93 | 0.88 |
| Selected features (n=5) of Cleveland heart disease dataset | | | | | | | |
| 10-fold | 92.60 | 91.65 | 92.72 | 95.80 | 91.48 | 0.93 | 0.85 |
| 7-fold | 92.14 | 91.30 | 92.12 | 94.65 | 91.40 | 0.92 | 0.84 |
| 5-fold | 91.80 | 90.16 | 91.54 | 94.30 | 91.22 | 0.92 | 0.83 |

Table 4. Performance of ET classifier on different CV and subset feature spaces on S2

| Selected features (n=7) of Hungarian heart disease dataset | | | | | | | |
| --- | --- | --- | --- | --- | --- | --- | --- |
| **K-Fold CV** | **Accuracy** | **Sensitivity** | **Specificity** | **AUC** | **Precision** | **F1-score** | **MCC** |
| 10-fold | 96.81 | 97.24 | 96.30 | 98.16 | 97.12 | 0.97 | 0.94 |
| 7-fold | 95.55 | 97.04 | 94.59 | 97.40 | 97.12 | 0.96 | 0.93 |
| 5-fold | 95.33 | 97.15 | 94.58 | 96.90 | 97.02 | 0.95 | 0.92 |
| Selected features (n=6) of Hungarian heart disease dataset | | | | | | | |
| 10-fold | 98.04 | 97.21 | 98.15 | 98.95 | 98.03 | 0.98 | 0.95 |
| 7-fold | 96.60 | 97.24 | 96.07 | 98.06 | 97.28 | 0.97 | 0.94 |
| 5-fold | 96.05 | 96.76 | 95.62 | 96.81 | 96.92 | 0.96 | 0.93 |
| Selected features (n=5) of Hungarian heart disease dataset | | | | | | | |
| 10-fold | 96.80 | 97.20 | 96.25 | 97.80 | 97.12 | 0.97 | 0.94 |
| 7-fold | 95.42 | 96.30 | 95.18 | 97.65 | 96.42 | 0.96 | 0.93 |
| 5-fold | 95.30 | 97.16 | 94.54 | 97.35 | 96.02 | 0.95 | 0.92 |
